# High Throughput Analysis of Golgi Structure by Imaging Flow Cytometry

**DOI:** 10.1038/s41598-017-00909-y

**Published:** 2017-04-11

**Authors:** Inbal Wortzel, Gabriela Koifman, Varda Rotter, Rony Seger, Ziv Porat

**Affiliations:** 1 Dept. of Biological Regulation, the Weizmann Institute of Science, Rehovot, Israel; 2 Dept. Of Molecular Cell Biology, the Weizmann Institute of Science, Rehovot, Israel; 3 Dept. of Life Sciences Core Facilities, the Weizmann Institute of Science, Rehovot, Israel

## Abstract

The Golgi apparatus is a dynamic organelle, which regulates the vesicular trafficking. While cellular trafficking requires active changes of the Golgi membranes, these are not accompanied by changes in the general Golgi’s structure. However, cellular processes such as mitosis, apoptosis and migration require fragmentation of the Golgi complex. Currently, these changes are most commonly studied by basic immunofluorescence and quantified by manual and subjective classification of the Golgi structure in 100–500 stained cells. Several other high-throughput methods exist as well, but those are either complicated or do not provide enough morphological information. Therefore, a simple and informative high content methodology should be beneficial for the study of Golgi architecture. Here we describe the use of high-throughput imaging flow cytometry for quantification of Golgi fragmentation, which provides a simple way to analyze the changes in an automated, quantitative and non-biased manner. Furthermore, it provides a rapid and accurate way to analyze more than 50,000 cells per sample. Our results demonstrate that this method is robust and statistically powerful, thus, providing a much-needed analytical tool for future studies on Golgi dynamics, and can be adapted to other experimental systems.

## Introduction

The Golgi complex regulates various processes including vesicles transport, protein modification and sorting, and lipid biosynthesis^1, 2^. Although the basic functions of the Golgi are conserved through evolution, its structural organization varies between species. In yeast, individual cistranes or stacks of cistranes are functional and dispersed in the cytosol^3, 4^. In higher eukaryotes, the Golgi is composed of stacks of flattened cistranes that are connected by tubular bridges and localized at the perinuclear region^5^. This structure is termed intact/compact Golgi and is actively maintained during interphase by: (i) Golgi structural proteins, such as Golgins^6^, (ii) regulatory kinases^7^, (iii) constant membrane trafficking from the endoplasmic reticulum (ER)^8^ and (iv) cytoskeleton motors that control the structure as well as the perinuclear localization of the Golgi apparatus^9^.

In the past, electron microscope images gave the notion that the Golgi is a static organelle. However, it was later demonstrated that the Golgi is rather dynamic, and changes its morphology in response to physiological (mitosis, apoptosis and migration)^10–13^, and pathological processes (cancer, neurological diseases)^14–16^. During mitosis, division of the Golgi complex between the two daughter cells, occurs in a two-step process. First, the Golgi is fragmented into isolated stacks (partial fragmentation) at the G2/M border. This is followed in metaphase by a further fragmentation into a large number of vesicles, which are dispersed throughout the cytosol during anaphase as well (full fragmentation). Finally, the Golgi is rebuilt into one complex at telophase^17^. Blocking Golgi fragmentation attenuates cell cycle progression, and is considered as mitotic entry ‘check-point’^18^. Partial Golgi fragmentation also occurs during directional migration, as it is required for reorientation and reassembly of the Golgi towards the leading edge of the cell^11, 12^. Indeed, when Golgi structural proteins such as Golgin-160 and GMAP210 were knocked out, the Golgi in those cells underwent permanent fragmentation, which prevented the orientation of the Golgi and migration^19, 20^. In apoptosis, the Golgi undergoes fragmentation as a part of the organized destruction of the cells. Although many of the factors in the apoptotic process are shared with the mitotic fragmentation process^21, 22^, the former process is irreversible and leads to a complete Golgi destruction.

The intact structure of Golgi is often disrupted under pathological conditions as well. The presence of fragmented Golgi in tumors was first demonstrated by electron microscopy^23^, and suggested to induce cancer cell survival by affecting the activity of anti-apoptotic kinases (recently reviewed at^15^). Although the cancer-dependent Golgi fragmentation is known for a long time, the molecular mechanisms inducing it have only been studied recently. Thus, it was shown that the Rab proteins localize to different parts of the Golgi, and interact with Golgins. During tumorigenesis, the expression of the Rabs is increased and leads to aberrant interactions that destabilize the Golgi structure^24–26^. Golgi fragmentation induces substantial changes in the structural organization of the glycosyltransferase family in the Golgi, thus, leading to formation of cancer specific epitopes^23, 27^. Finally, fragmentation of the Golgi is a common occurrence in neurodegenerative diseases such as Alzheimer’s disease, Amyotrophic lateral sclerosis (ALS), Parkinson’s disease and others (recently reviewed at^28, 29^). Many of these diseases have deficient axonal transport, which leads to accumulation of protein in the cytoplasm. This aggregation of proteins may promote the disassembly of the Golgi apparatus^28, 29^. However, further investigation is required to fully understand the Golgi fragmentation during these processes.

Methods to study Golgi fragmentation can be classified into two categories: small scale and large scale. Among the small scale the classical method is basic immunofluorescence (IF), in which the cells are fixed and stained for a Golgi marker protein. Then, 100–500 cells are viewed by a fluorescence microscope, and subjectively categorized into intact or fragmented Golgi. In addition, there are a few methods that utilizes living cells, for example; Fluorescence Recovery After Photobleaching (FRAP)^30^ of the Golgi^31^, in which, cells are stably transfected with a tagged Golgi protein that can diffuse through the Golgi membranes and recover after the photobleaching as long as the Golgi tubular connection exists. By measuring the recovery curve of 10–20 cells, the Golgi connectivity and structure can be deduced. A different approach is to deplete living cells from the Golgi by laser nano-surgery, and follow them by time-lapse and electron microscopy^32^. However, this method is more suitable for the study of Golgi biogenesis, rather than Golgi organization. Several methods were published for a large-scale analysis of the Golgi structure. One example for such a method is the staining of cis, medial and trans Golgi, followed by imaging and analyzing 2000 cells for their Golgi structure^7^. This method, though able to analyze a larger number of cells, requires 3 different staining and stable transfections. Other high-content methods analysis of the Golgi that were recently published include the use of a direct stochastical optical reconstruction microscopy (dSTORM)^33^, machine learning^34^ and flow cytometry-based measurements (Pulse-shape analysis - PulSA) of a fluorescently labeled molecule, to evaluate if the Golgi distribution is concentrated (intact) or spread (fragmented)^35, 36^. While all of the methods described above are valid, their use is rather complicated, or lack morphological information and therefore, additional, simpler high content methods to follow Golgi fragmentation should be beneficial.

In this paper we describe the development of a method to detect and quantify changes in the Golgi structure in a non-subjective manner using Imaging Flow Cytometry (IFC). In IFC, cells in suspension are passed through the instrument in a single file, where they are illuminated by a set of lasers and LEDs. The images are captured by a dedicated CCD camera, which enables up to 10 fluorescent channels and bright-field (BF) images^37–39^. This is done at a rate of up to 5000 cells/sec, allowing acquisition of a statistically robust sample in a short time. By using IFC we were able to rapidly collect thousands of cells, and then use a simple analysis template that we developed for quantification of the Golgi fragmentation in a non-biased way. This provides a way to objectively detect the changes of the Golgi structure of the population in a statistically robust manner. Moreover, we tested and verified our method by examining various conditions that mediate Golgi disassembly. Thus, our method provides an important and convenient tool for the future study of this essential process.

## Results

### Development of IFC as a tool to study Golgi structure

**Figure 1:**
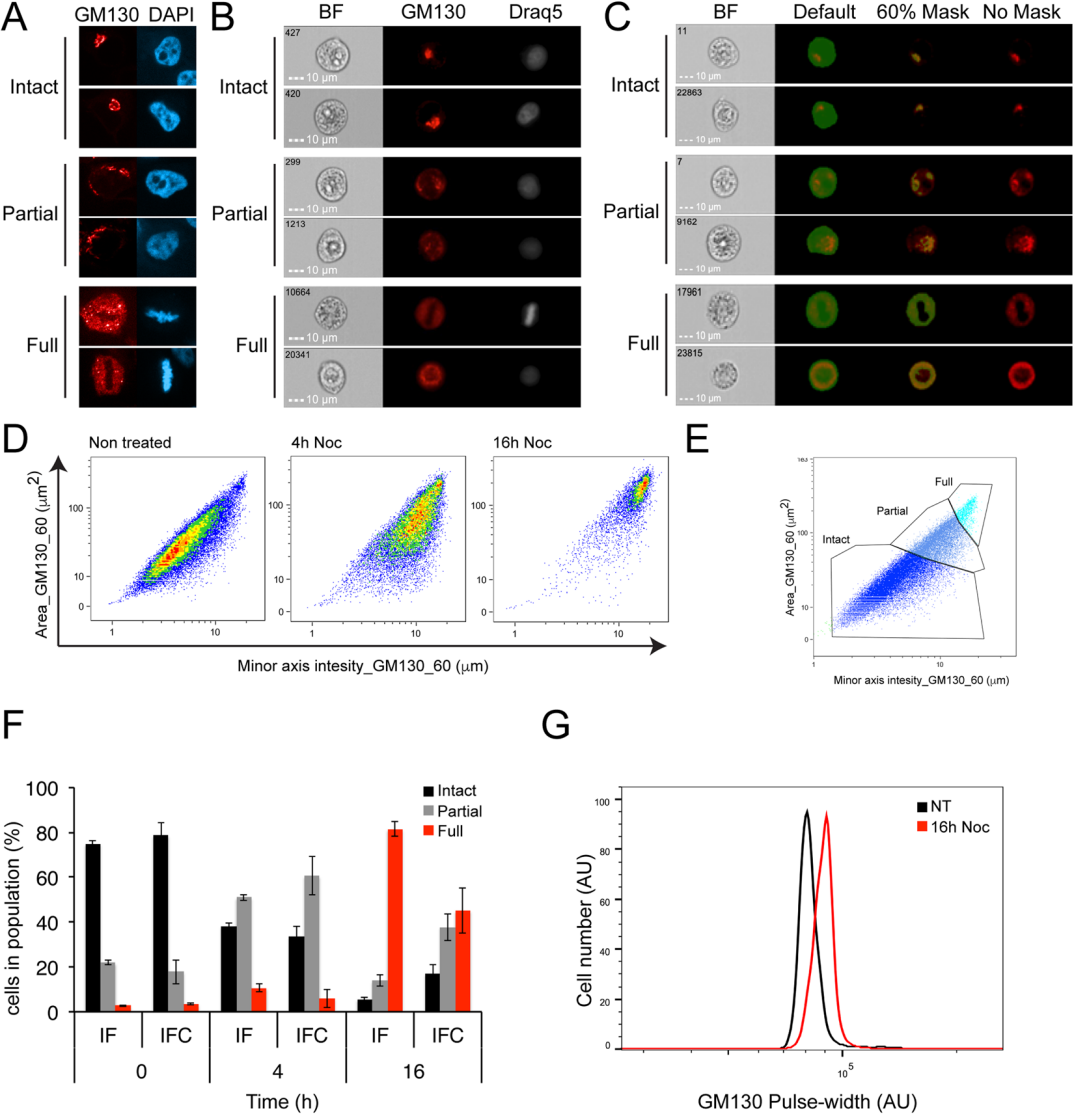
Definition of Golgi positive staining and gating for Golgi morphologies. HeLa cells were fixed and stained for the Golgi marker GM130 (Red) and the DNA stains DAPI (blue) or Draq5 (White), then at least 100 cells were imaged by a confocal spinning disc microscope (**A,F**) or, 3 × 10^4^ cells were imaged by the Imaging Flow Cytometer ImageStreamX mark II (**B–F**) or BD LSR II (**G**), (BF = bright field, Noc = Nocodazole, IF = Immunofluorescence, IFC = Imaging Flow Cytometry). **(A,B)** Representative images of the three possible morphologies of the Golgi (**A**) IF (**B**) IFC. **(C)** A mask of the 60% highest pixels (green) was defined on top of the Golgi staining to differentiate between the Golgi structures. **(D)** HeLa cells were treated with nocodazole (0.3 μM) for 4 or 16 hours or left untreated. The Golgi structure was analyzed according to two parameters referring to the top 60% intensity pixels of the GM130 staining (using the Threshold_60 mask): The area of staining (Y axis), and the intensity weighted, narrowest dimension of the best fit ellipse of the mask (X axis). The cells were plotted on a density dot-plot. **(E)** Intact Golgi shows small area and small width, while fragmented Golgi has a large area and a round morphology. Three populations were defined as intact, partially and fully fragmented Golgi. **(F)** Comparison between IF and IFC for quantification of Golgi morphologies distribution upon Nocodazole treatment, as described above. Results are average ± SEM (IF, n = 4, IFC, n = 3). **(G)** HeLa cells were treated with 0.3 μM nocodazole for 16 hours or left untreated. Representative histograms of Pulse-shape analysis (PulSA) values (the width of the GM130 signal) for GM130 of two independent experiments (Shown is the single width of the Golgi staining).

The Golgi complex is a dynamic organelle that changes its morphology during several cellular processes. In order to study the changes in the structure of the Golgi, we chose HeLa cells, since their Golgi structure is mostly intact, but the changes in its morphology are readily detected in cycling, apoptotic, or migrating cells, and widely used in the field of Golgi fragmentation^40^. HeLa cells were fixed and stained for the Golgi marker GM130 and a DNA stain, followed by analysis with a confocal spinning microscopy (Fig. 1A) and IFC (Fig. 1B). All three Golgi morphologies (intact, partial and full fragmentation) were detected in the population by a visual inspection (Fig. 1A,B). In order to find the best parameters to distinguish between the different morphologies, we first needed to delineate the Golgi staining. In IFC, analysis is done by calculating a set of parameters, termed “features”, done on a defined area of interest, termed “mask”. We therefore created several masks of the highest intensity pixels to identify the Golgi staining, and calculated all available features of the Golgi stain (e.g. area, intensity, diameter). Next, we manually selected ~100 cells of each morphology and using the IDEAS analysis software, we calculated the RD value (Fisher’s discriminant) to distinguish between the combination of masks and features that gives the best statistical separation between the chosen populations^41–43^. The best features to distinguish between the morphologies were the area and the minor axis intensity, both of them calculated for a threshold mask of the 60% highest pixels of the Golgi staining (Fig. 1C, see Methods for detailed description).

In order to test the above parameters on the whole population, we plotted a density dot-plot with the minor axis intensity of the Threshold 60% mask as the X-axis, and the area of the Threshold 60% mask as Y-axis. Most of the HeLa cells population was localized at the bottom of the graphs, where both the area and the axis length were low; this combination reflects a state in which most of the population has an intact Golgi (Fig. 1D, non-treated). We then induced fragmentation of the Golgi by nocodazole, which interferes with microtubule association and therefore arrest cells in the prometaphase stage of the cell cycle^44, 45^. This was done for 4 and 16 hours, to induce partial and full fragmentation of the Golgi, respectively. After 16 hours of nocodazole, the population shifted to the upper-right end of the graph, representing cells with full fragmentation of the Golgi (Fig. 1D, 16 h). On the other hand, the 4 hours nocodazole treatment induced only partial fragmentation of the Golgi, and indeed, the population was concentrated at the center of the graph (Figs 1D, 4h). We defined three gates around the center of each treatment, delineating the three morphologies, further to additional visual inspection (Fig. 1E).

### Comparison of IFC measurements to IF and PulSA

To validate our measurements, we compared them to the classical method of IF imaging and manual counting (Fig. 1F), and to PulSA measurements of Golgi fragmentation (Fig. 1G). The distribution in the population of cells with either intact, partially or fully fragmented Golgi was comparable between the confocal images and our IFC analysis in the non-treated control and 4 hours treatment (Fig. 1F), confirming the validity of our method. The distribution in the 16 hours treated sample was different between the methods, which we attribute to the great loss of cells from the slide in the IF method. The IFC sample preparation prevents this problem (by taking into account all the cells), thus demonstrating higher accuracy. The same results were observed in COS7 cells (see Supplementary Fig. S1). We then analyzed the same samples by PulSA and followed the GM130 distribution. The nocodazole treated sample demonstrated a shift (Fig. 1G), which is indicative of Golgi fragmentation as described before^36^. However, while the PulSA can indicate if there is a change of Golgi cellular distribution, its measurements can neither identify the degree of fragmentation (partial or full), nor can it quantify accurately the percentage of Golgi fragmentation sub-populations in the sample.

### Using IFC to study mitosis-dependent Golgi fragmentation and reassembly

**Figure 2:**
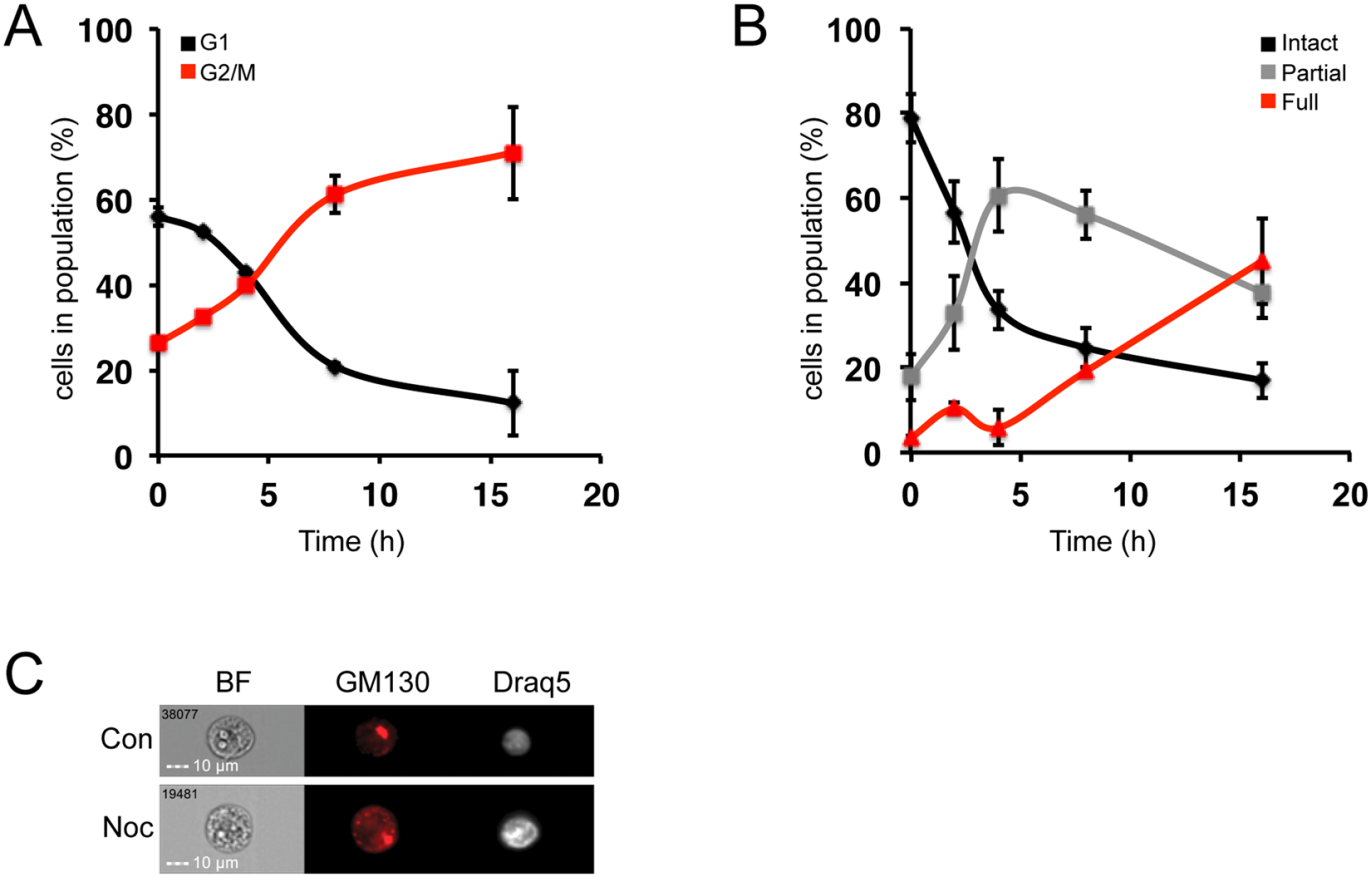
**Nocodazole-induced mitotic arrest and Golgi fragmentation**﻿**.﻿** HeLa cells were treated with 0.3 μM nocodazole for the indicated times, then fixed and stained for GM130 and Draq5. **(A)** G1 and G2/M stages of the cell cycle were analyzed according to the Draq5 intensity. **(B)** The 3 populations of the Golgi structure were analyzed at each time point. **(C)** Representative images of cells from each treatment.

We then went on to utilize this set of parameters in the study of mitotic Golgi fragmentation. First, we used the nocodazole-treated HeLa cells described above to gain a better insight into the kinetics of Golgi fragmentation during cell cycle progression using the method developed. As expected, in resting cells (time 0), most of the HeLa cell population was at G1, while nocodazole treatment reduced the G1 population and increased the G2/M population (Fig. 2A). The changes in cell cycle were accompanied by gradual fragmentation of the Golgi complex. At time 0, the population demonstrated mostly intact Golgi, while 2 and 4 hours after nocodazole treatment, there was a substantial increase in the partially fragmented population that was accompanied by a decrease in the intact Golgi. Longer nocodazole treatments of 8 and 16 hours induced a significant increase in the fully fragmented population (Fig. 2B,C). These results show that the nocodazole-induced Golgi fragmentation occurs in two phases, first, partial fragmentation, which is observed in the short treatment, and then full fragmentation observed in the long treatment. Although we used a previously reported nocodazole concentration^18, 46, 47^, in other studies a higher concentration of nocodazole (up to 10 μM) was used^48, 49^. To confirm the validity of our results, we repeated the experiments for the 16-hour time point with both 0.3 μM and 10 μM nocodazole. However, no difference between the different concentrations in either our analysis or the PulSA methods was detected (see Supplementary Fig. S2).

**Figure 3:**
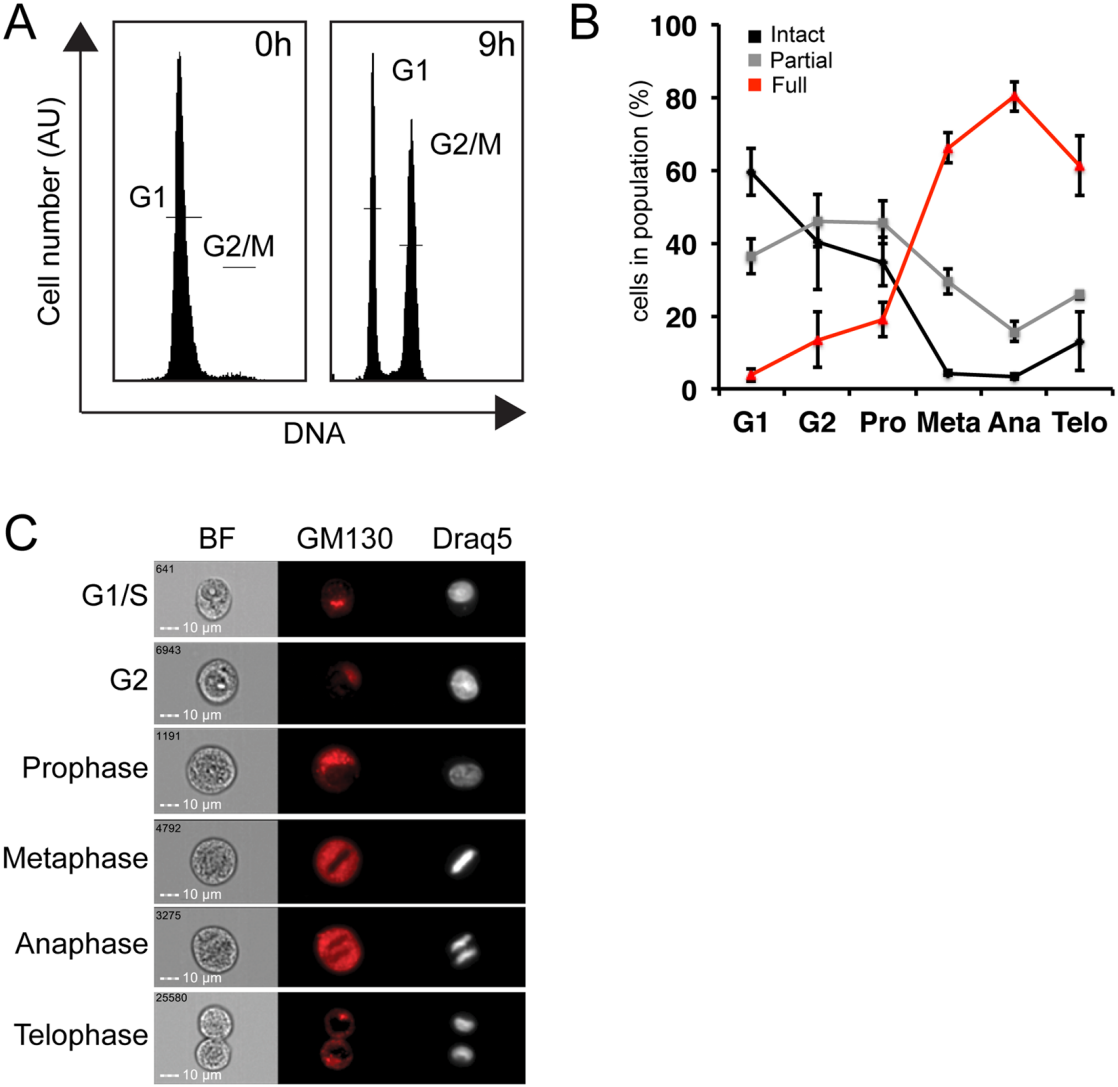
In mitosis, the Golgi fragmentation starts at Prophase, and the Golgi assembly starts at Telophase. HeLa cells were synchronized with the double thymidine block protocol. The cells were fixed at the G1/S border, or released for 9 hours, at the peak of mitosis, and fixed. The cells were stained for GM130 and Draq5. **(A)** Cell cycle histogram of the cells from the G1/S border (0 h) or at the peak oh mitosis (9 h). **(B)** The 3 populations of the Golgi structure were analyzed at each phase of the cell cycle. **(C)** Representative images of cells from the cell cycle phases.

Mitotic Golgi fragmentation is a reversible process, in which the Golgi is broken at the beginning of mitosis, and is rebuilt at its end. We then tested this breakdown and the rebuilt of the Golgi during mitosis after the release from double thymidine G1/S arrest (Fig. 3A). Nine hours after the release, the G2/M population was further divided into G2 and the sub-mitotic phases (prophase-telophase) according to cellular and DNA staining morphology, as previously described^46^. Using our method, we then analyzed the percentage of cells with intact, partially and fully fragmented Golgi at each phase. As expected, at the G1/S border most of the population demonstrated an intact Golgi (Fig. 3B,C). Cells at late G2 (the population was fixed at the mitotic peak, thus the cells that are in G2 are mostly late G2) and prophase demonstrated an increase in the partially fragmented population, and a decrease of the intact population. As mitosis progresses to metaphase and anaphase, the cells demonstrated mainly fully fragmented Golgi. At telophase, the end of mitosis, we can detect the rebuilt of the Golgi apparatus as seen by the reduction of the fully fragmented population and the increase in the intact and partial populations (Fig. 3B,C). Similar results were observed with a different Golgi protein, GRASP65 staining (see Supplementary Fig. S3), thus indicating that this analysis can be applied for staining for other Golgi marker proteins as well. Therefore, our method accurately quantifies the physiological changes of the Golgi structure, both disassembly and reassembly, and therefore can be used to shed a new light on the dynamic process of Golgi fragmentation.

### Detecting Golgi fragmentation during non-mitotic processes

**Figure 4:**
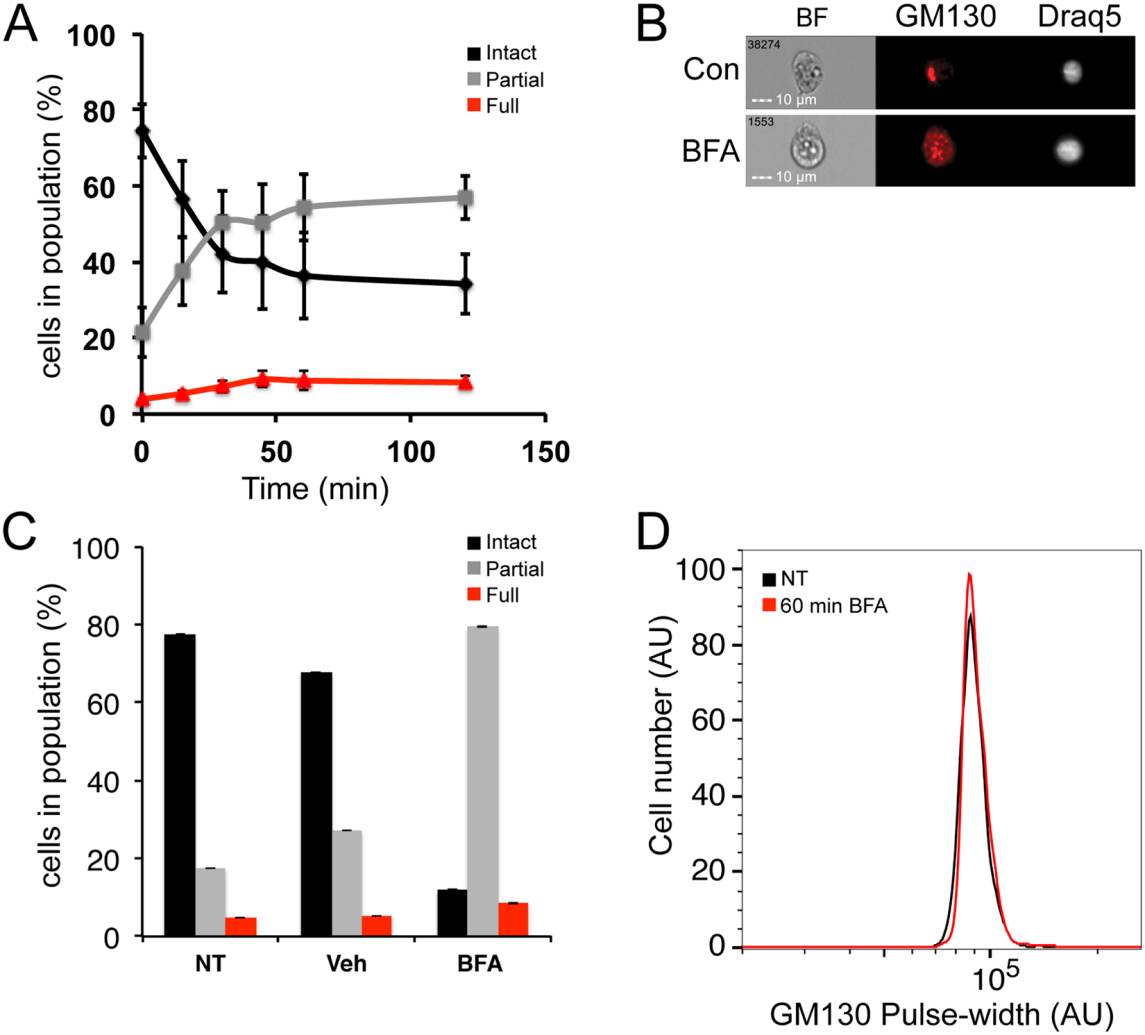
Brefeldin A induced rapid Golgi fragmentation. HeLa cells were treated with 0.2 μM BFA for the indicated times, then fixed and stained for GM130 and the Draq5. **(A)** Intact, Partially and fully fragmented populations were analyzed by IFC at each time point. **(B)** Representative IFC images of cells from non-treated and 60 min BFA treatment. **(C)** HeLa cells were treated with 0.2 μM BFA or vehicle for 60 min or left untreated. Cells were fixed and stained for GM130 and DAPI. Cells were imaged by confocal spinning disc microscope, manually counted and classified into the 3 Golgi populations. More than 150 cells were counted for each treatment, n = 2. **(D)** HeLa cells were treated with 0.2 μM BFA for 60 min or left untreated. Representative histograms of PulSA values (the width of the GM130 signal) for GM130 of two independent experiments.

Both nocodazole and the release from double thymidine block used above induce a mitosis-dependent Golgi fragmentation. In order to study whether IFC can be used to study Golgi fragmentation during other processes, we first tested Brefeldin A (BFA), which induces Golgi fragmentation by blocking membrane trafficking from the ER to the Golgi^50^. HeLa cells were treated with BFA for up to 2 hours, and then fixed and stained with anti GM130 antibody. At time 0, the cell population contained mainly an intact Golgi, while after 2 hours, most cells contained partially fragmented Golgi (Fig. 4A,B). The BFA-dependent fragmentation was rapid, and reached a plateau after 60 min. Interestingly, BFA-induced a full fragmentation only in a very small portion of the treated cell population, indicating that BFA affects only the first, and not the second stage of the fragmentation. Longer treatment of BFA for up to 6 hours, did not induce further fragmentation of the Golgi in any of the treated cells (data not shown). We then verified our results using both IF and PulSA methods. While IF counting confirmed our IFC analysis (Fig. 4C), these changes were not detected by PulSA (Fig. 4D). Furthermore, a ten-fold increase in the BFA concentration yielded the same results (see Supplementary Fig. S4). We further verified our results with another Golgi protein - Giantin^51^, because GM130 partitions to the intermediate compartment and not to the ER upon BFA treatment, unlike other Golgi markers. Similarly to GM130 staining, the IFC analysis of Giantin demonstrated partial Golgi fragmentation in both concentrations. Interestingly, in this case, the PulSA analysis was able to detect Golgi fragmentation as well (see Supplementary Fig. S4).

**Figure 5:**
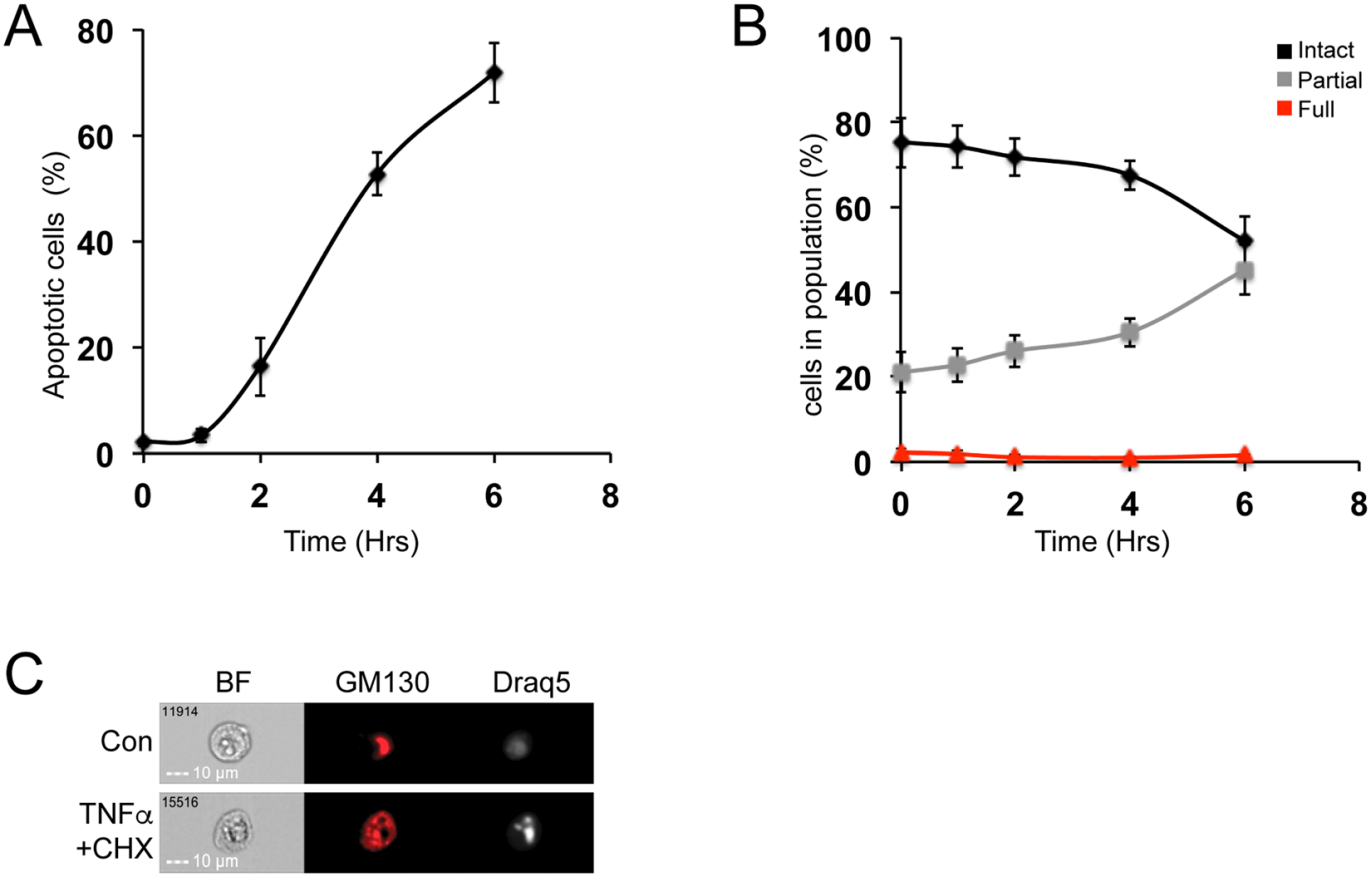
Demonstration of TNFα/Cycloheximide induced Golgi fragmentation in apoptosis. HeLa cells were treated with 25 ng/ml TNFα and 1 μg/ml Cycloheximimde (CHX) for the indicated times, then fixed and stained for GM130 and Draq5 and analyzed by IFC. **(A)** Percentage of apoptotic cells was determined by the BF contrast and the area of the brightest pixels of the Draq5 staining (for details see materials and methods). **(B)** Intact, Partially and fully fragmented populations were analyzed at each time point. **(C)** Representative images of cells from each treatment.

We next examined whether our method could also detect the apoptosis-dependent Golgi fragmentation. To do so, we induced apoptosis in HeLa cells using a combination of TNFα and Cycloheximide treatment^52–54^. To identify the apoptotic cells in the examined population, we used IFC as previously described^55^; cells with high contrast and low area of the DNA staining (condensed DNA) were considered apoptotic (for details see Materials and Methods). In the untreated cells, we detected less than 3% apoptotic cells, while 6 hours after TNFα and Cycloheximide treatment more than 70% of the cells were apoptotic (Fig. 5A). When we then followed the changes in the structure of the Golgi apparatus, we found a steady decrease in fragmentation in the intact population, and an increase in the partially fragmented population (Fig. 5B,C). Many factors and proteins regulate both mitotic and apoptotic Golgi fragmentation (e.g GRASP65 regulates both processes, but is phosphorylated during mitosis, and ubiquitinated during apoptosis^21^). However, in agreement to previous studies^17^, our results show that full fragmentation of the Golgi apparatus is detected mainly during mitosis.

### Studying Golgi fragmentation in cancer

**Figure 6:**
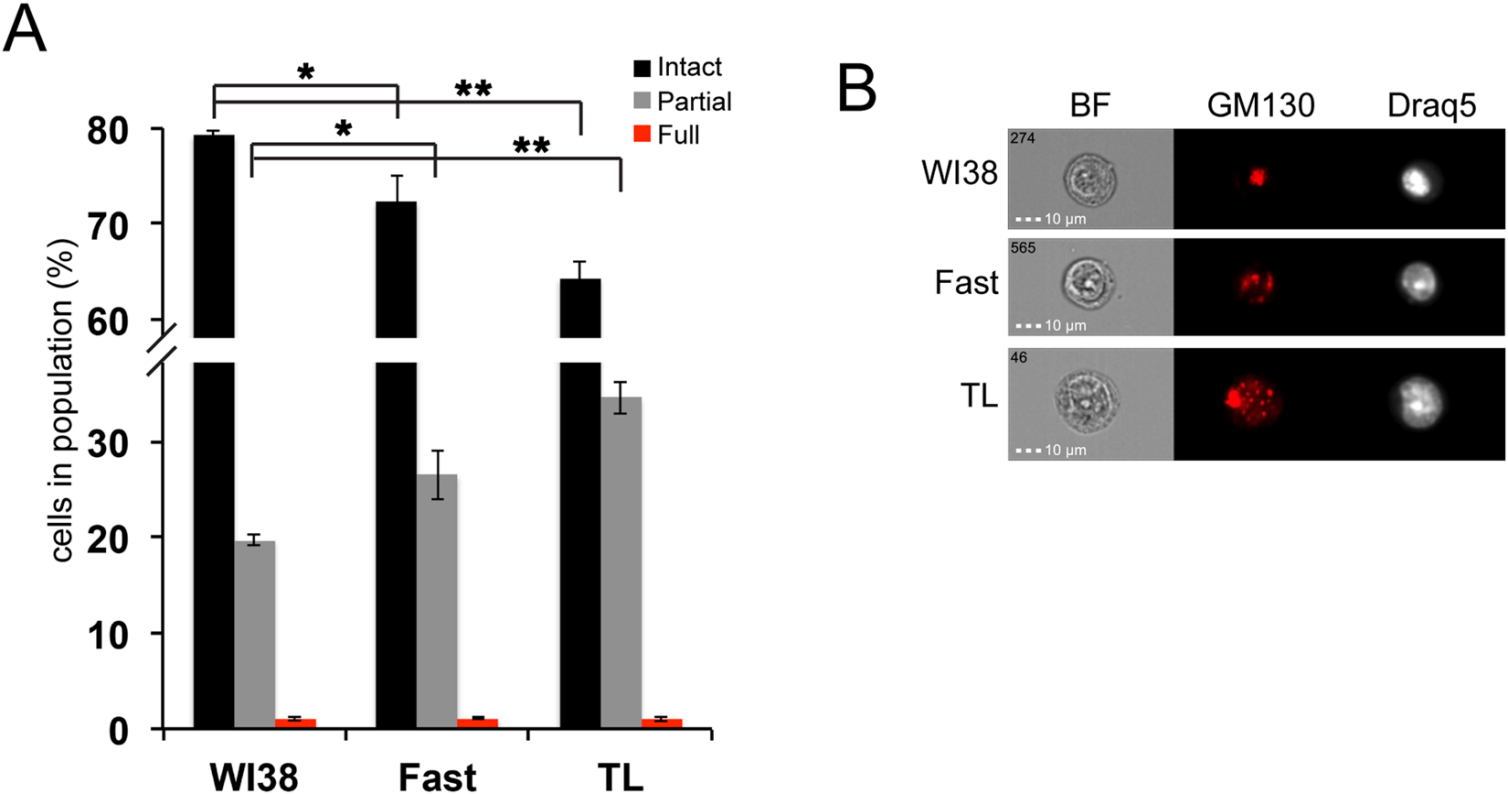
Tumorigenic transformation is accompanied by increased levels of Golgi fragmentation. WI-38, WI-38/hTERT/H-Ras/GSE (Fast) and their derived tumor lines cells (TL) were fixed with Ethanol-HBSS (80–20%, 1 × 10^6^ cells). The cells were stained for GM130 and Draq5 and analyzed by IFC **(A)** The 3 populations of the Golgi structure were analyzed for each cell line. **(B)** Representative images of cells from each cell line.

Finally, we wanted to test our Golgi structure quantification method in a pathological system. Cancer dependent fragmentation is a well-described phenomenon, which is thought to increase survival and protection from anti-apoptotic kinases in the tumor cells (recently reviewed in ref. 15). To study the cancer dependent Golgi fragmentation we utilized an *in-vitro* transformation model that was previously described^56^. This model included WI-38, normal human fibroblasts, WI-38/hTERT/H-Ras/GSE, immortalized WI-38 that were transformed by a concomitant expression of H-Rasv^12^ and wild type p53 inactivating peptide (GSE56), and two tumor-derived lines (TL) that were generated by subcutaneous injection of the WI-38/hTERT/H-Ras/GSE into nude mice^57^. We first defined the Golgi fragmentation populations for WI38 cells by nocodazole treatment, as we have done for the HeLa cells (data not shown). Once the populations were defined, we stained the WI-38, WI-38/hTERT/H-Ras/GSE and TL cells for GM130 and quantified the stages of Golgi fragmentation in these cell-lines (Fig. 6). We found that WI38 cells demonstrated mainly an intact Golgi, while less than 20% of the population showed partially fragmented Golgi. The WI-38/hTERT/H-Ras/GSE cells displayed an increase in the partially fragmented Golgi population compared with the WI-38, while the WI-38/hTERT/H-Ras/GSE derived TL had the largest population of partial fragmentation (more than double of the WI-38). This model demonstrates the effect of cancer dependent Golgi fragmentation. Specifically, we show here that in cells showing higher tumorogenic potential, a larger percentage of the population displays a fragmented Golgi. Overall, our results show that by measuring morphological features of the Golgi staining, different fragmentation stages can be detected and quantified in a rapid, accurate and an objective way.

## Discussion

Golgi fragmentation occurs in a variety of physiological and pathological conditions, and it is often used as readout for various biological processes (e.g mitosis, viral infection, neurodegenerative diseases)^58–60^. Accurate quantification of this phenomenon is therefore essential in studying numerous biological questions. Current methods for assessment of Golgi fragmentation include mainly basic immunofluorescence of the Golgi followed by counting of approximately 100 cells. These are then manually classified into intact, partially and fully fragmented Golgi, which is laborious and user-biased. Recently, several, much needed, high content methods have been described as well. Most of them are imaging-based methods, with the benefit of being high-content, high throughput and/or automated analysis. However, the disadvantages of these methods are that they require either establishment of stable over-expression cell lines, sophisticated microscopy techniques, or development of advanced image-analysis methods^7, 32–34, 61^. Other high-throughput methods utilize conventional flow cytometry for evaluation of signal distribution within the cell. However, the resolution of this method limits the accurate identification of the different Golgi morphologies^33–36^ and it lacks the morphological features of microscopy. Moreover, while PulSA measurements may be useful for large sub-cellular compartments^62^, changes in a small compartment such as the Golgi are more difficult to quantify (compare Figs 1G, 4B, and S4B,E). Thus, the current methods are either difficult to use or lack essential information. Hence, we set to develop an easier and straightforward method for this purpose.

We present here the development of a high-throughput, automated method to quantify changes of the Golgi structure in a large population of cells. For this purpose we utilized the AMNIS ImageStreamX, an imaging flow cytometer, which allows rapid collection of a large set of high quality images and consequent automated analysis. To detect the Golgi, cells were stained for several Golgi markers, and Draq5 to detect the DNA. In the analysis template we developed, we were able to define three populations of cells with intact, partially fragmented or fully fragmented Golgi, by measuring the length of the minor axis and the area of the Golgi staining. However, as we demonstrate here, there is no clear structural separation between the three morphologies of the Golgi, but rather a gradual change in the structure. This further demonstrates that the manual assessment of the Golgi structure is problematic, as there are many in-between morphologies of the Golgi. We utilized nocodazole-treated samples, resulting in mainly partial fragmentation (after 4 hours) followed by mostly full fragmentation (after 16 hours) to define the three Golgi morphologies, in addition to visual inspection of the gated cells. Hence, employing gates that were verified and used throughout the analysis ensured the objectivity of our measurements.

Using our analysis template, for an untreated population of HeLa, COS7 and WI-38 cells more than 70% of the population demonstrated an intact Golgi, less than 5% were fully fragmented (representing the mitotic population of cells in the culture), and the rest had a partially fragmented Golgi. By using various agents and chemicals that induce disassembly of the Golgi apparatus, we verified that these measurements could accurately follow changes in the Golgi structure for the total population. Full fragmentation of the Golgi apparatus is also known as ‘mitotic haze,’ and indeed, we detected an increase in the fully fragmented Golgi population only in conditions that induced mitotic dependent Golgi fragmentation (e.g nocodazole and cell synchronization). Importantly, we detected an increase of the partial fragmentation in the tumorogenic cell lines. This was not a result of high percentage of dividing cells, as there was no significant change in either cell cycle (data not shown), or the fully fragmented population.

The high number of cells acquired allows analysis of rare populations, as specific stages of mitosis (e.g., anaphase cells represent less than 1% of the population). The ability to simultaneously collect several fluorescent channels enables further classification of the acquired population. We used here the nuclear image to distinguish between interphase and dividing cells, including the specific mitotic stages (which is not available in conventional flow cytometry methods, as PulSA), and to identify apoptotic cells. As there are total of 10 available fluorescent channels, this can be further used to identify a very specific population, often required for immunological applications, for example, or to be combined with other parameters. We applied the ‘find the best feature’ approach^41–43^ to find the best feature to define the Golgi morphologies using the manufacturer analysis software, IDEAS. Recently, several reports combined the imaging abilities of the ImageStream with enhanced analysis by utilizing machine learning approaches^63, 64^. It could be interesting to add these image analysis techniques in future studies.

We utilized the unique abilities of IFC to rapidly acquire tens of thousands of high quality multispectral images, collected in a uniform manner. We further developed a simple analysis platform to quantify the Golgi fragmentation, based on Golgi proteins staining. This is done in an automated, unbiased manner, eliminating the need for the laborious manual acquisition and inspection of the acquired images and avoiding the inter-user variability in image analysis. In addition, the high-throughput acquisition and analysis allows statistically robust conclusions, which is more difficult to achieve with conventional microscopy. Moreover, while the other methods mostly classified the Golgi structure into either intact or fragmented, we were able to further characterize the fragmentation into full and partial fragmentation. We verified our method for various biological and chemical conditions that induce changes in the Golgi structure. Our findings also show novel features of the fragmentation processes such as the lack of full fragmentation in cancer and apoptosis. Moreover, we verified our measurements in several cell lines, providing evidence that our method could be easily adapted for different biological systems. We demonstrated in this work compelling evidences for accurately measuring changes in the Golgi structure, in an objective, rapid and high-throughput way, which may serve as a beneficial tool for the cell biology field.

## Materials and Methods

### Cell Culture

HeLa and COS7 cells were cultured in Dulbecco’s modified Eagle’s medium (DMEM), supplemented with 2 mM L-glutamine, 1% Pen/Strep and 10% fetal bovine serum (FBS). Primary human embryonic lung fibroblast, WI-38, WI-38/hTERT/H-Ras/GSE^56^ and their derived tumor lines were grown in MEM supplemented with 10% FCS, 2 mM L-glutamine, 1 mM sodium pyruvate and antibiotics. Cells were maintained at 37 °C in a humidified atmosphere of 95% air and 5% CO_2_.

### Antibodies and Reagents

Thymidine, nocodazole, Cycloheximide, Brefeldin A and DAPI were purchased from Sigma-Aldrich (St. Louis, MO, USA); hTNFα from Peprotech (Rocky Hill, NJ) and Draq5 from BioStatus, (Leicestershire, UK). Rabbit monoclonal α-GM130 Ab, Rabbit α-Giantin from Abcam (Cambridge, UK); Rb polyclonal α-GRASP65 was kindly gifted from Prof. Sima Lev; Alexa Fluor® 568 from Invitrogen (Carlsbad, CA).

### Cell synchronization

Cells were synchronized at the G1/S boundary by the double thymidine block^47^. In short, HeLa cells were treated with 2.5 mM thymidine in DMSO, washed twice with PBS, grown for 8 h in regular medium, treated again with 2.5 mM thymidine for 16 h and then washed with PBS. This marks time 0, after which the cells were grown under regular conditions. HeLa cells were also synchronized at prometaphase using 0.3 or 10 μM nocodazole for 16 h.

### Apoptosis Induction and Brefeldin A treatment

HeLa cells were induced for apoptosis using 25 ng/ml hTNFα and 1 μg/ml Cycloheximide for up to 6 h. For induction of Golgi fragmentation with Brefeldin A, HeLa cells were treated with 0.2 μg/ml or with 2 μg/ml for up to 120 min.

### Multispectral imaging flow-cytometry (IFC) analysis

Cells were fixed by methanol fixation and resuspended in a blocking-permeabilization solution (2% Albumin bovine serum (BSA), 0.1% Triton X-100 in PBS^−/−^, 5 min, 23 °C). The cells were then incubated with the primary Ab to detect the Golgi (1 h, 4 °C, gentle agitation), washed once with PBS^−/−^ and then incubated with Alexa Fluor® 568-conjugated secondary Ab (1 h, 4 °C, gentle agitation), and Draq5 for DNA staining. Cells were imaged using multispectral imaging flow cytometry (ImageStreamX mark II imaging flow-cytometer; Amnis Corp, Seattle, WA, Part of EMD Millipore). Approximately 5 × 10^4^ cells were collected from each sample and data were analyzed using image analysis software (IDEAS 6.2; Amnis Corp). Images were compensated for fluorescent dye overlap by using single-stain controls. Cells were gated for single cells, using the area and aspect ratio features, and for focused cells, using the Gradient RMS feature, as previously described^65^. Cells were gated for G2/M based on Draq5 intensity. The G2/M population was further gated for prophase, prometaphase and mitotic (metaphase-telophase) populations using 2 parameters: the bright detail intensity of Draq5 staining (intensity of localized bright areas, subtracted for the local background), and the area of the 50% highest intensity pixels of the Draq5 staining (defined by the Threshold_50 mask). The different mitotic stages were further defined by the nuclear morphology, as previously described^46^. To quantify the Golgi fragmentation, two features were eventually chosen based on the GM130 staining: Minor Axis Intensity (the intensity weighted narrowest dimension of the ellipse of best fit) and Area (the number of microns squared within a mask). These were calculated on the Threshold_60 mask that includes the 60% highest intensity pixels of the GM130 staining. These two features were plotted as a bivariate plot and the different Golgi morphologies were gated according to visual inspection. To validate the gating strategy, Nocodazole treated samples (in which the Golgi is either partially or fully fragmented in a time dependent manner) were used. For quantification of apoptotic cells, single, focused cells were plotted for the contrast of the bright field channel vs. the area of the 50% highest intensity pixels of the Draq5 staining (defined by the Threshold_50 mask). Cells with high contrast and low area (condensed) of the DNA staining were considered apoptotic^55^.

### Immunofluorescence microscopy

Cells were fixed in 4% paraformaldehyde in PBS (20 min, 23 °C), and incubated with 2% Albumin bovine serum (BSA) in PBS (15 min, 23 °C), followed by permeabilization with Triton X-100 (0.1% in PBS, 5 min, 23 °C). The fixed cells were then incubated with the primary Abs (1 h, 23 °C), washed three times with PBS and incubated with fluorescent secondary Ab (1 h, 23 °C), and DAPI. Slides were visualized by a Spinning Disk Confocal Microscopy (x60 magnification Zeiss, Jena, Germany). Background correction and contrast adjustment of raw data images were performed, using Photoshop (Adobe, CA, USA).

### Flow cytometry – PulSA

The cells were prepared as described for IFC, and analyzed by LSR II (Becton Dickinson, NJ, USA). For Pulse-shape analysis (PulSA), the area (A), width (W) and height (H) of Golgi marker staining was collected. Data was analyzed using FLowJo V.10 (FlowJo, LLC, USA).

### Statistical Analysis

Data are expressed as mean ± S.E. Statistical evaluation was carried out using functional analysis and Student’s T-test (two-tailed) to test for differences between the control and experimental results. Values of *p* < 0.05 were considered statistically significant.

## Electronic supplementary material

Supplementary Data
