# Supplementary material for: High Throughput Analysis of Golgi Structure by Imaging Flow Cytometry
Source: Sci Rep. 2017 Apr 11;7:788. doi: 10.1038/s41598-017-00909-y (PMC5429768; doi:10.1038/s41598-017-00909-y)
Supplement: Supplementary file 1 — Supplementary Data [file 41598_2017_909_MOESM1_ESM.pdf]

High Throughput Analysis of Golgi Structure by Imaging Flow Cytometry

Supplemental Data

Inbal Wortzel[1], Gabriela Koifman[2], Varda Rotter[2], Rony Seger[1] and Ziv Porat[3*]

[1]Dept. of Biological Regulation, [2]Dept. Of Molecular Cell Biology, [3]Life Sciences Core Facilities, the Weizmann Institute of Science, Rehovot, Israel.

* Corresponding author: Ziv.porat@weizmann.ac.il

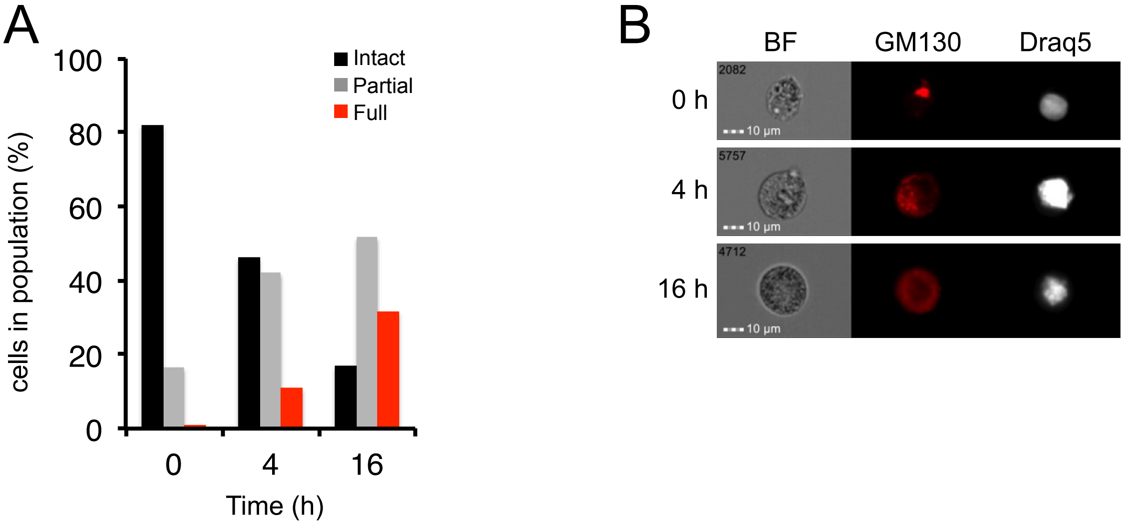

Figure S1: analysis of COS7 cells for Golgi fragmentation by IFC
COS7 cells were treated with 0.3 µM nocodazole for the indicated times, then fixed and stained for GM130 and Draq5. **(A)** The 3 populations of the Golgi structure were analyzed at each time point. **(B)** Representative images of cells from each treatment.

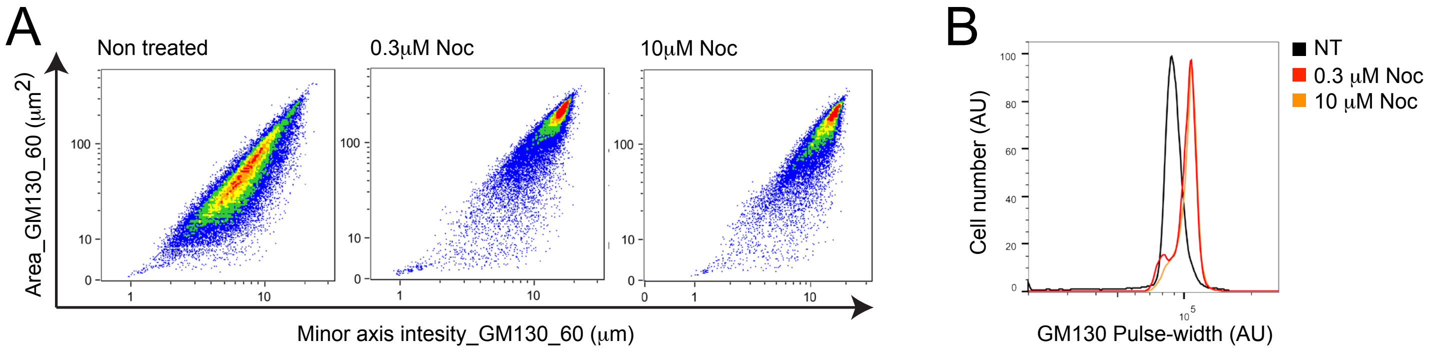

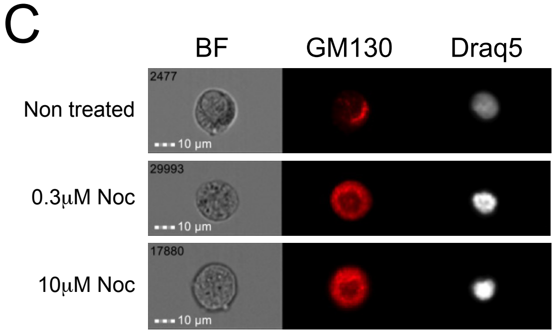

Figure S2: Increasing nocodazole concentration has no further effect on Golgi fragmentation
HeLa cells were treated with 0.3 or 10 µM nocodazole for 16 h, then fixed and stained for GM130 and Draq5. **(A)** IFC analysis of the Golgi structure for each treatment. **(B)** Representative histograms of PulSA values (the width of the GM130 signal) for each treatment of two independent experiments (Shown is the single width of the Golgi staining). **(C)** Representative images of cells from each treatment.

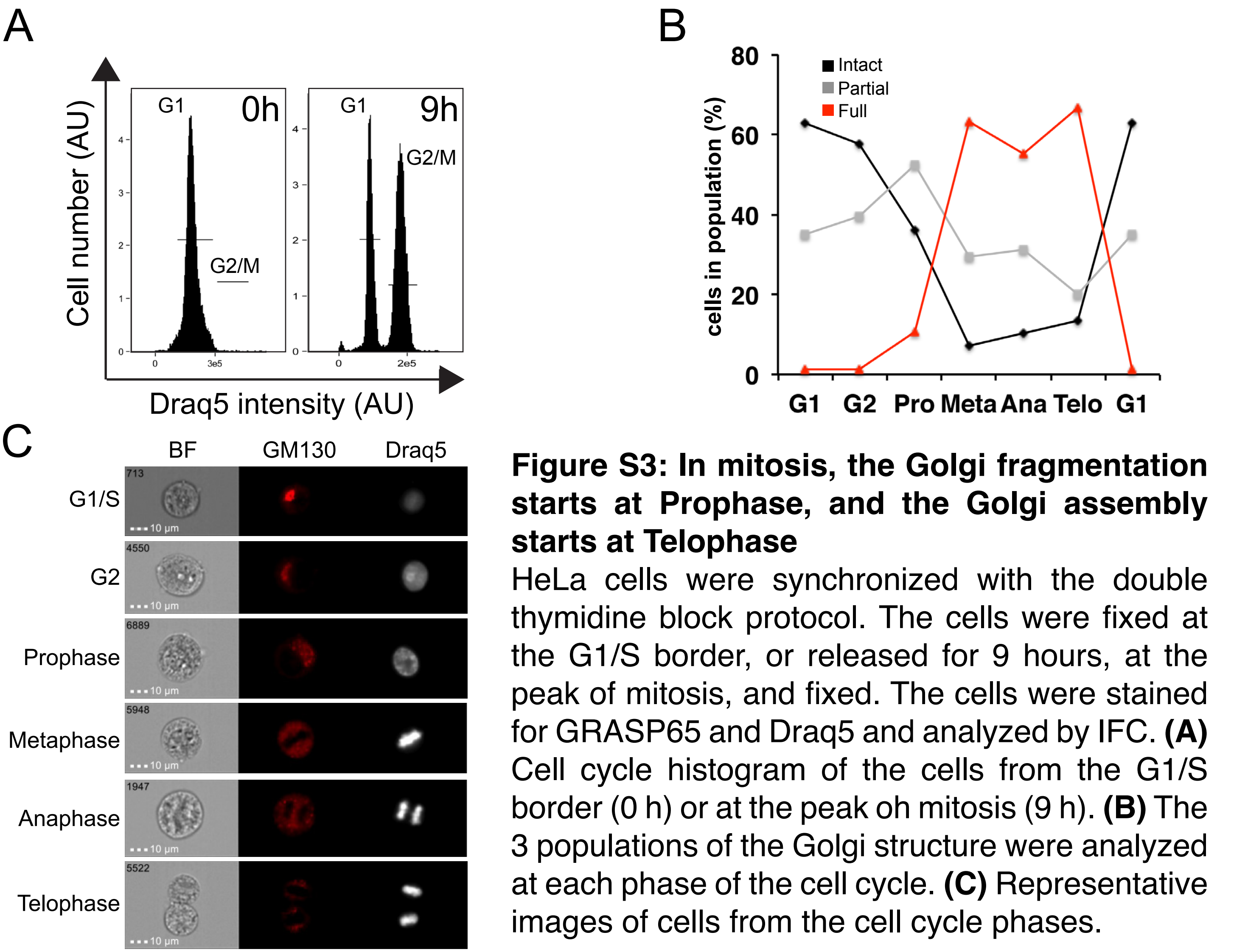

Figure S3: In mitosis, the Golgi fragmentation starts at Prophase, and the Golgi assembly starts at Telophase

HeLa cells were synchronized with the double thymidine block protocol. The cells were fixed at the G1/S border, or released for 9 hours, at the peak of mitosis, and fixed. The cells were stained for GRASP65 and Draq5 and analyzed by IFC. **(A)** Cell cycle histogram of the cells from the G1/S border (0 h) or at the peak oh mitosis (9 h). **(B)** The 3 populations of the Golgi structure were analyzed at each phase of the cell cycle. **(C)** Representative images of cells from the cell cycle phases.

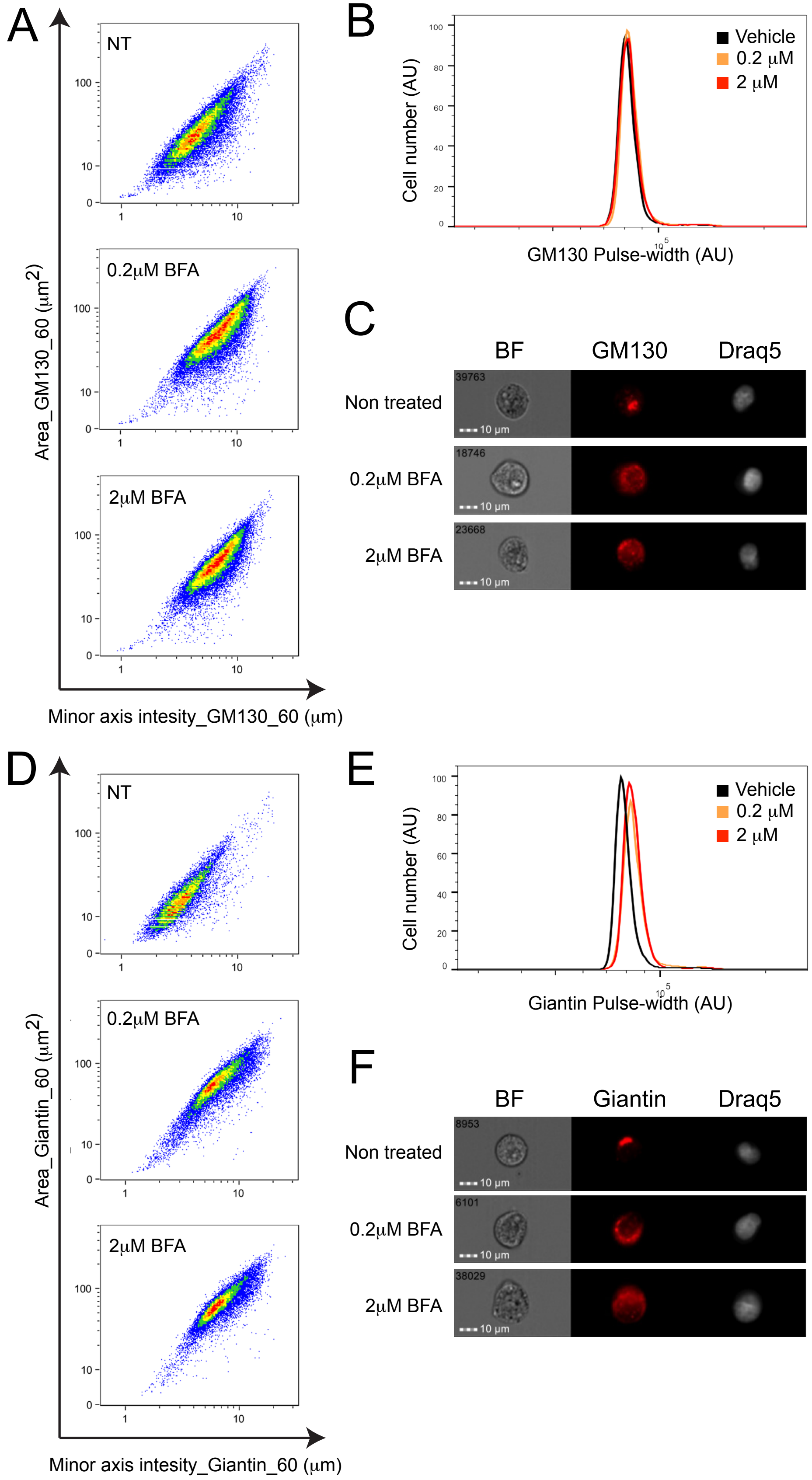

Figure S4: Increasing BFA concentration has no further effect on Golgi fragmentation
HeLa cells were treated with 0.2 or 2 µM BFA for 60 min, then fixed and stained for GM130 **(A-C)** or Giantin **(D-F)** and Draq5. **(A, D)** IFC analysis of the Golgi structure for each treatment. **(B, E)** Representative histograms of PulSA values (the width of the signal) for each treatment of two independent experiments (Shown is the single width of the Golgi staining). **(C,F)** Representative images of cells from each treatment.
